# An allied research paradigm for epidemiology research with Indigenous peoples

**DOI:** 10.1186/s13690-019-0353-1

**Published:** 2019-05-20

**Authors:** Denise Jaworsky

**Affiliations:** 1 Northern Medical Program, University of Northern British Columbia, 3333 University Way, Prince George, BC V2N 4Z9 Canada; 2 Management and Evaluation, Health Sciences Building, University of Toronto, Institute of Health Policy, 155 College Street, Suite 425, Toronto, ON M5T 3M6 Canada; 3 Department of Medicine, University of British Columbia, 2775 Laurel Street, 10th Floor, Vancouver, BC V5Z 1M9 Canada

**Keywords:** Allyship, Epidemiology, Methodology, Cross-cultural research, Colonialism, Reconciliation

## Abstract

**Background:**

There is no shortage of epidemiology research describing the ill health of Indigenous peoples in Canada and globally and many of these studies have had negative repercussions on Indigenous communities. However, epidemiology can also be a helpful tool for supporting the health and health services of communities. This paper challenges the reader to consider the harms of epidemiology which essentialize Indigenous communities as sick and in need of help. It then discusses, from the perspective of a settler physician and clinical epidemiology student, how we may be able reconcile the field of epidemiology research with the needs of Indigenous communities. In doing so, it describes an allied research paradigm for epidemiology.

**Results:**

Although qualitative research has been substantially informed by critical feminist theories, uptake in quantitative research has been sparser. It is even more rare for Indigenous methodologies to be used to inform quantitative research. This paper is written from a personal perspective, reflecting on the author’s prior experiences as well as existing literature on critical feminist theory and Indigenous methodologies, to describe an allied research paradigm. This allied research paradigm follows an ontology that explores the subjectivity within epidemiology and the influence of the positionality of the researcher. It follows an epistemology that understands that knowledge can be generated through many ways including, but not limited to statistical analyses. It follows an axiology that research aims to affect social change and improve the lives of the communities participating in the research. It follows a methodology that is participatory and empowers community partners to meaningfully contribute to statistical research. This allied research paradigm, which makes no claims to universality, describes several important principles: reconciliation, relationships, perspective, positionality, self-determination and accountability.

**Conclusion:**

Researchers who wish to engage in research in allyship with Indigenous communities must understand the colonial history embedded in health research, commit to a process that honours meaningful relationships with community partners, and carefully consider the implications of their work.

## Background

The landscape of epidemiology relating to Indigenous populations continues to evolve, along a pathway of prepositions – from “on” to “with” to “by.” As Indigenous self-determination in epidemiology research continues to strengthen, it is important to reflect critically on the role of the ‘allied’ researcher. As members of the dominant group, we must question how we can engage in epidemiology work in Indigenous health without perpetuating ongoing colonial structures. I draw on critical feminist theory and Indigenous methodologies to articulate the beginning of an ‘allied research paradigm’ for epidemiology in Indigenous health. While written for epidemiology research, I encourage academics in other fields, particularly those who have not yet engaged community in their work, to think about how to apply to your own discipline. I offer this paper not as a prescription or solution, but as a launching point for discussion. I see this as a beginning rather than a complete paradigm with the understanding that as my relationship with my research and the people involved grows, so will my articulation of an allied research paradigm.

Knowledge is political, and by extension, so is research. Thus, who we are as researchers and as people will inform and influence the work that we do and the knowledge that we produce. Our positionality dictates the questions that we ask, the methods that we use, the way we interpret our findings and what we do with our findings once the knowledge has been generated. My positionality is situated in my multiple identities as a settler physician in the speciality of internal medicine, as a PhD student in clinical epidemiology at the University of Toronto, as a (half-)Japanese-Canadian and as a person living in a region of Northwestern British Columbia, Canada, which is the territory of the *Tsm’syen (Tsimshian)* peoples. Although I identify with my Japanese heritage, I live my day-to-day life as an essentially white upper-middle class physician and woman and benefit immensely from the associated privilege and power [1–3]. My identification with the dominant group is likely two-fold, through the cultural assimilation that my family experienced during the Japanese internment in Canada and also the perceived demarginalization of Japanese Canadians following the Canadian government’s redress of these human rights abuses [4]. Although I do not identify as an activist researcher, my research motivation is driven by a desire to create social change that will improve population health and I recognize that my relationship with my research introduces subjectivity into my work.

### Past and ongoing harms to Indigenous peoples

There is no shortage of medical literature on the health ‘problems’ of Indigenous peoples in Canada and globally, and I argue that as health researchers we must be much more cognizant of how our research can influence the dominant culture’s perceptions of Indigenous peoples and also Indigenous peoples’ understandings of their own identity. Health epidemiology, particularly as applied to health policy has a longstanding history of essentializing Indigenous people as sick or in need of help [5]. In doing so, it often creates a false dichotomy of the healthy white population and the sick Indigenous population, particularly when using statistical health indicators in the absence of historical, social and cultural contextualization [6]. Even well-intentioned work can have unintended harmful repercussions and portray Indigenous peoples as deviant, diseased and dependent while setting dominant societal standards as the norm and positioning Indigenous realities as needing to achieve that norm. This perpetuates stereotypes and is often conducted without any tangible benefit to the researched communities [7, 8]. Furthermore, this negative portrayal of Indigenous communities in academic literature can be used to justify paternalism and threaten efforts of Indigenous self-determination [9].

Unfortunately, this reductionism occurs frequently in health research, and when epidemiology research places Indigenous heritage into statistical models, in the absence of contextualization, resultant findings contribute to the portrayal of Indigenous peoples as deviant, diseased and dependent. For example, a study on leaving the hospital against medical advice for people who use injection drugs described that, “factors positively associated with leaving the hospital against medical advice included recent injection drug use, Aboriginal ancestry, leaving on weekends and welfare check day” [10]. In this sentence, Indigenous people have been identified as exhibiting the deviant behaviour of leaving the hospital against medical advice and that this behaviour is also shared with people who have recently injected drugs and people receiving a recent social assistance check. There is no contextualization of factors that may affect Indigenous peoples’ tendency to leave against medical advice, such as experiences of racism within healthcare or perceptions that care is not culturally safe and no suggestion of how this research could improve the health of Indigenous peoples. Although the authors may not have intended to assign this negative reputation to Indigenous peoples, they made no demonstrated effort to avoid perpetuating dominant culture’s negative perceptions towards Indigenous peoples.

Issues of individual and community consent are also very important. Indigenous heritage is often used as a covariate in analyses, rarely with the explicit consent of individuals or communities to look at Indigenous heritage as contributing to deviant behaviour or disease. This issue of consent has been previously described as it relates to anonymous unlinked HIV seroprevalence studies of Indigenous peoples where blood collected for another purpose was later tested for HIV and the findings were used to generate data on rates of HIV prevalence among Indigenous peoples [11]. Research conducted in this manner has no direct benefit to Indigenous peoples and leads to further discrimination against Indigenous peoples.

In addition to the potential negative repercussions of research, the research process itself can be colonizing. Māori scholar, Linda Tuhiwai Smith, articulates that “the ways in which scientific research is implicated in the worst excesses of colonialism remains a powerful remembered history for many of the world’s colonized peoples [7].” Racist assumptions of intellectual superiority, devaluing of Indigenous ways of knowing, the use of research findings to support lands dispossession and threaten self-determination, and the western concept of intellectual property and ownership of knowledge are ways in which research itself can be colonizing. Research and the quest to better understand the world does not, however, need to be colonizing and I will highlight some of the ways that Indigenous scholars have begun to decolonize the research process.

With this understanding of how epidemiology has been problematic for Indigenous populations, I move forward trying to understand how, as a clinical epidemiologist, I can mitigate the harms of my work. In doing so, I present an outline for an allied research paradigm and six key principles for consideration based on my own experiences. My hope is that I can contribute to epidemiology work that promotes health equity and use what has been described as “credibility tactics” to leverage well-accepted epidemiology methods to address social, political and colonial determinants of health [12].

### Approach

I write from the perspective of a settler quantitative graduate student. The target audience includes other quantitative and mixed methods researchers as well as other health researchers who aim to ally with Indigenous communities. I reflect on a discussion of existing paradigms, theories and methodologies, my personal journey as a researcher and interactions with colleagues and co-researchers. I write through a lens of reconciliation, guided by critical feminist theories, Indigenous methodologies and community-based participatory methodologies. I also include stories from my research journey. Through this process, I actively engage in reflexivity and honour the importance of storytelling in Indigenous knowledge sharing. As a settler, I cannot understand the full cultural significance or protocols of storytelling beyond what I have read in texts. Cree/Saulteaux scholar, Margaret Kovach, explains that “stories are vessels for passing along teachings, medicines, and practices that can assist members of the collective [13].” It is with this intention that I have included my own stories.

Reflexivity includes reflection on my own positionality within my research and on how I can learn from my experiences [14]. It also involves careful consideration of an epistemology, axiology and ontology that are concordant with my own worldviews but also privilege Indigenous voices in research methodology. Reflexivity is a way to constantly examine our work and apply a critical lens so that our work does not inadvertently fortify oppressive structures and colonial agendas [15]. In being reflexive, I hope to continue the process of decolonizing my own mind, my approach to research and the academic spaces that I exist in. The development of this allied research paradigm was also informed by discussions with colleagues, friends and mentors who are Indigenous researchers, allied researchers, community members and students. A similar method has been previously described by Shawn Wilson [7] in his articulation of an Indigenous research paradigm.

### Allyship

Articulation of an allied research paradigm first requires an understanding of the concept of “allyship.” Allyship cannot be explained with a one-size-fits-all universal definition. The gay rights literature is rich with discussions of ally identity, which includes the complexity of ‘insider-outsider’ position as well as commonalities and differences between allies and the communities with whom they offer their solidarity [16]. For example, allies share a political stance, values and a sense of connectedness with these communities, but they must go through a process of proving their alignment with the values and interests of the particular community or movement they align with [17]. While the gay rights literature provides many insights into allyship that can be applied to relationships with other communities, the nature of the allyship relationship varies based on the parties involved. Anishinaabe-kwe scholar, Lynn Gehl presents a bill of responsibilities for allies. This bill describes principles of responsible allyship, several of which include awareness of oppressive structures, discussing privilege, and not taking resources of the oppressed group [18].

I describe principles of allyship based on my experiences with Indigenous friends, colleagues and communities, and the relationships that I have formed and my interpretation of how they would want me to act as an ally. I describe my journey towards being an ally in research to Indigenous communities in Canada who are affected by HIV/AIDS. This is a journey that I began over ten years ago, but it has taken a long time for me to thoughtfully consider what this means. Before turning to my own experiences, I first draw from understandings of allyship in published literature.

Ashley Heaslip, a settler physician and ally in health research to Indigenous peoples, describes allyship as a concept “imbued with the notion of cultivating, building and strengthening relationships between two differing individuals, groups or communities based on respectful, meaningful and beneficial interactions [19].” Allyship is not a state of permanence or a credential that can be achieved. It is based on relationships that are context-specific, responsive and require continuous renegotiation. An ally to an oppressed group has an ongoing responsibility to demonstrate that they continue to be an ally.

In the context of research with Indigenous peoples, Johnson and Madge [20] describe allies as collaborative partners to Indigenous communities, supporting their self-determination and pursuing research aligned with community priorities. For settlers and migrants, allying ourselves with Indigenous peoples necessitates a resistance to imperialism and colonialism. This is particularly challenging in the context of health research where research *on* Indigenous peoples has furthered and continues to further oppressive colonial agendas. Allies in research to Indigenous peoples engage in research *with* Indigenous peoples and support research *by* Indigenous peoples.

A key consideration in allyship is the question of who determines whether one is an ally or a well-intentioned ‘white saviour’ [21]. Some may consider themselves an ally if they act as a bridge between academia and the community, or because they help to build research capacity in the communities that they research, but have we taken the time to ask Indigenous groups what they value in an ally? In respect of their self-determination, should our merit as an ally not be judged by the standards of the group we hope to ally with? Indigenous activist, Jessica Danforth, warns that many will declare themselves to be an ally without consulting the community itself [22]. That is not to say that allies are not valued. The privilege they experience, once recognized, can also be used to take a stand against oppression. [23, 24]*.*

Rather, critically reflecting on our role as an ally is an ongoing process that must be imbedded in the work that we do, and we must be responsive to our own reflections as well as those of the communities to which we are accountable. Indigenous scholar and scientist, Kim TallBear, articulates the importance of this in allyship when she affirms that “a researcher who is willing to learn how to ‘stand with’ a community of subjects is willing to be altered, to revise her stakes in the knowledge to be produced [25].”

Allyship also extends well beyond a particular project or community that researchers engage with. As a member of a dominant group, to become an ally is more than just working or showing support to address a particular ‘problem’ experienced by another group or redress wrongs as a reaction to ‘white guilt’ [26]. It involves a deeper understanding of the structures that underlie our privilege and contribute to the oppression of other groups of people [27] along with a commitment to dismantle these structures [28]. Within epidemiology, this can begin with a critical reflection of how our research questions, our methods, and the institutions where we conduct our work contribute to the oppression of the populations for whom the research is intended to benefit. This includes embarking in the intellectual and emotional journey towards awareness of our contribution to ongoing injustices [26], for example through the deficit-based portrayal of oppressed populations. Rather than looking only to identify poor health outcomes and predictors of disease, we must look to the strengths of communities, culture, land, and language and how these can be predictors of health and wellness. Reframing writing so that strengths are identified, described, quantified and used to address health disparities can be a step away from a deficit-based approach. At the same time, we can also redirect our gaze from the Indigenous ‘other’ to the role our own practices, histories and colonial structures play in perpetuating health inequity [29]. When working collaboratively with Indigenous peoples, allyship can begin with educating ourselves about settler colonialism, Indigenous culture and values, historical and current political relationships and acts of resistance [21]. It can begin by opening our academic spaces to be inclusive of other paradigms, theories and ways of knowing.

### An allied research paradigm

#### Paradigms in epidemiology and possible alternatives

Most quantitative research lends itself to a positivist paradigm that is guided by the scientific method and assumes that objectivity and reason can uncover a single truth or reality. Accordingly, positivist assumptions guide understandings of reality (ontology), what is considered knowledge (epistemology) and values (axiology). In a positivist ontology, reality exists as a single, objective reality. In a positivist epistemology, knowledge is generated through the scientific methods and is reproducible and verifiable. In a positivist axiology, knowledge inquiry is value-free [30]. Methodology, or the ways that researchers discover more about a reality, is guided by ontology, epistemology and axiology and together, these four concepts can be used to construct a paradigm.

Numerous authors [31–34] challenge this positivist paradigm within epidemiology and argue that we may better serve the health needs of marginalized groups through a re-evaluation of how knowledge is produced through epidemiology. They call for a more comprehensive consideration of theory within epidemiology. In doing so, they turn to the social sciences to provide guiding theories, such as critical feminist theory [35]. As an example of an alternate paradigm, critical feminist theory challenges assumptions of a single objective (ontology) truth and views the nature of reality as one that is socially, culturally, historically and politically constructed and thus in flux as the structures that create power change. Knowledge is generated (epistemology) such that it can be used to empower people and transform their lives. Researchers’ values (axiology) align with those of social justice and a commitment to dismantle oppressive structures [30].

In his book, *Research Is Ceremony: Indigenous Research Methods*, Shawn Wilson [7] articulates an Indigenous research paradigm which honours Indigenous knowledge systems and worldviews and sees research as a sacred pursuit of knowledge. In an Indigenous research ontology, multiple realities may exist and through research, the researcher forms a relationship with a reality [7]. In an Indigenous research epistemology, the world is seen as a web of relationships and knowledge exists in the context of these relationships. An Indigenous axiology is built on principles of relational accountability such that “the methodology needs to be based in a community context (be relational) and has to demonstrate respect, reciprocity and responsibility (be accountable as it is put into action) [7].” He also turns to tools within participatory action research that can be useful within an Indigenous research paradigm and others such as Opelousas/Coushatta scholar, Bonnie Duran, have made key contributions to community-based participatory research methodologies [36]. The Indigenous research paradigm, as described by Wilson, is not specific to epidemiology research, nor is it the only Indigenous research paradigm. Many Māori scholars have used a Kaupapa Māori approach in epidemiology research which ensure that research is Māori initiated, defined and controlled [37, 38].

#### Development of an allied research paradigm

When I began graduate studies, I intended to apply an Indigenous research paradigm to my work on community engagement in epidemiology. As I continued to familiarize myself with works by Indigenous scholars such as Wilson and Tuhiwai Smith, I realized that Indigenous paradigms did not align with my own epistemology, axiology and ontology [7, 39]. Furthermore, it was clear that I was never the intended beneficiary of their work. I can learn about the historical and ongoing impacts of colonialism on the Indigenous peoples of Canada and strive to work in solidarity, but that is not my lived reality. However, I can strive to decolonize myself and my work and learn from the Indigenous scholars and mentors who share their teachings with me. Therefore, I do not feel that as an individual settler I can claim to work within an Indigenous Research Paradigm. As a part of a collective, led by Indigenous people who are guided by Indigenous axiologies and epistemologies, I could contribute to work from an Indigenous Research Paradigm, or as Māori researcher, Elana Curtis suggests, I could align my research approach to be *consistent with* an Indigenous paradigm [40]. These reflections led me to search for an allied research paradigm.

An allied research paradigm for epidemiology must challenge the dominant positivist assumptions of the field and allow for other ways of knowing. Recognizing that subjectivity exists within epidemiology and that the positionality of the researcher influences the research can allow for an ontology that can be reconciled with Indigenous research approaches. Madge et al. [41] argue that researchers bring subjectivity into quantitative research through the design of their data collection tools and analyses and purely objective epidemiology research does not exist. Covariates that are included in models can be selected by statistical methods or through clinical judgement. When clinical judgement is employed, researcher subjectivity, including their own experiences and the type of literature they choose to review can influence covariate selection. Defining categories for variables also introduces subjectivity into epidemiology. For example, including additional gender categories to the conventional dichotomy of ‘woman’ and ‘man’ may alter and enrich the analysis as well as provide findings that speak directly to individuals who are often invisible in epidemiology, such as trans individuals [42]. A researcher who works closely with trans communities may advocate for the inclusion of non-binary gender categories whereas one who does not may only include ‘woman’ and ‘man.’ These subjective experiences also influence how data are collected and how data collection instruments are designed.

An epistemology that is more inclusive of other forms of knowledge generation is necessary in an allied research paradigm. Knowledge may be held and shared through stories, videos, art and other modalities in addition to the peer-reviewed literature that is often seen by dominant academics as the only valid source of knowledge [43]. Even within peer-reviewed health literature, quantitative studies are often given more weight than qualitative studies. Oral histories and online videos may be useful in identifying research questions or important covariates. Honouring Indigenous ways of knowing is essential for researchers who aim to engage in meaningful collaborations with Indigenous communities. An allied research paradigm axiology should be guided by participatory approaches where the goal of the research is to affect social change and improve the health of communities and populations. Knowledge generation on its own is an insufficient end. Relational accountability, as articulated by Shawn Wilson [7], is also a key component of an allied axiology where the relationships formed during the research process are as important as the research output.

The ontology, epistemology and axiology of an allied research paradigm can be realized using a participatory methodology that is inclusive of epidemiology methods. Community-based participatory research (CBPR) is a field that has been evolving over the past several decades and developed on the premise that meaningful partnerships among academic researchers and communities are necessary to achieve health equity [44]. Community partners are meaningfully engaged throughout the research process and research is conducted with the ultimate goal of action based on the research findings. Within epidemiology, CBPR can be enacted through a process where epidemiologists share in the ownership of and power over a study and in return “make their research more relevant to communities by co-creating knowledge and generating meaningful data-driven change [32].” This process enables community members to contribute to setting the research agenda, designing data collection tools, interpreting findings and helping to translate findings directly to action aimed at improving population health. It involves capacity building to ensure that community partners are equipped with the necessary tools to meaningfully contribute, while also accommodating the use of scientific research methods. This process will lead to a shift in the types of questions that are asked such that they are more responsive to the knowledges important to communities and are able to drive policy based on community priorities.

Although CBPR methodologies are more common in qualitative research, community partners have also played key roles in quantitative research [38, 40, 45]. Benefits of CBPR methodologies to epidemiology include: 1) increased trust between researchers and communities, 2) increased data quantity and improved quality, 3) identification of new research questions based on community priorities and 4) improved knowledge translation and policy uptake of research findings [32]. One example of key community leadership in Indigenous epidemiology research in Canada is the First Nations and Inuit Regional Longitudinal Health Survey which was developed, conducted and owned by First Nations communities [9, 46]. It has been an instrumental source of credible data used to inform health policy decisions for Indigenous peoples in Canada.

Trawlwoolway scholar, Maggie Walter, and Métis scholar, Chris Anderson explain how Indigenous methodologies can be inclusive of quantitative methods [47]. They call for a distinction between quantitative methods (for example regression analysis) and the methodology that informs the entire research process. It is “the methodology, rather than the method of analysis, [that] contains the cultural, social, and consequently, political meanings of research process and practice [47].” Thus, quantitative methods, including the broad range offered in the field of epidemiology, can be used as tools within Indigenous quantitative methodologies.

Articulating an ontology, epistemology, axiology and methodology of an allied research paradigm provides a theoretical foundation, but how do we put this into practice? In the next section, I offer some suggestions that can be applied by epidemiologists working in allyship with Indigenous communities. I frame these suggestions in my own experiences and interpretations from the literature. Many of these can apply to a broad scope of research methods and designs, but as a clinical epidemiology graduate student, I present these for consideration by colleagues in my own field in the hopes of beginning to decolonize our research space.

### Important principles in an allied research paradigm

I first became involved in Indigenous health research through my mentors, Dr. Mona Loutfy (ally) and the late LaVerne Monette who was the Executive Director of the Ontario Aboriginal HIV/AIDS Strategy at the time when I knew her. When I first entered this field in 2007, I was an eager medical student who wanted to help Indigenous people with their health problems. Over the past decade, my perspective has shifted, and I problematize the ongoing oppressive structures of colonialism rather than the health of Indigenous peoples. I try to frame my research questions using this perspective, although admittedly this is challenging as I have been educated within a deficit-based dogma. It is the responsibility of allied researchers to challenge this deficit-based dogma and support the efforts of Indigenous colleagues who are reframing Indigenous health epidemiology [9, 37, 38, 46].

**Fig. 1:**
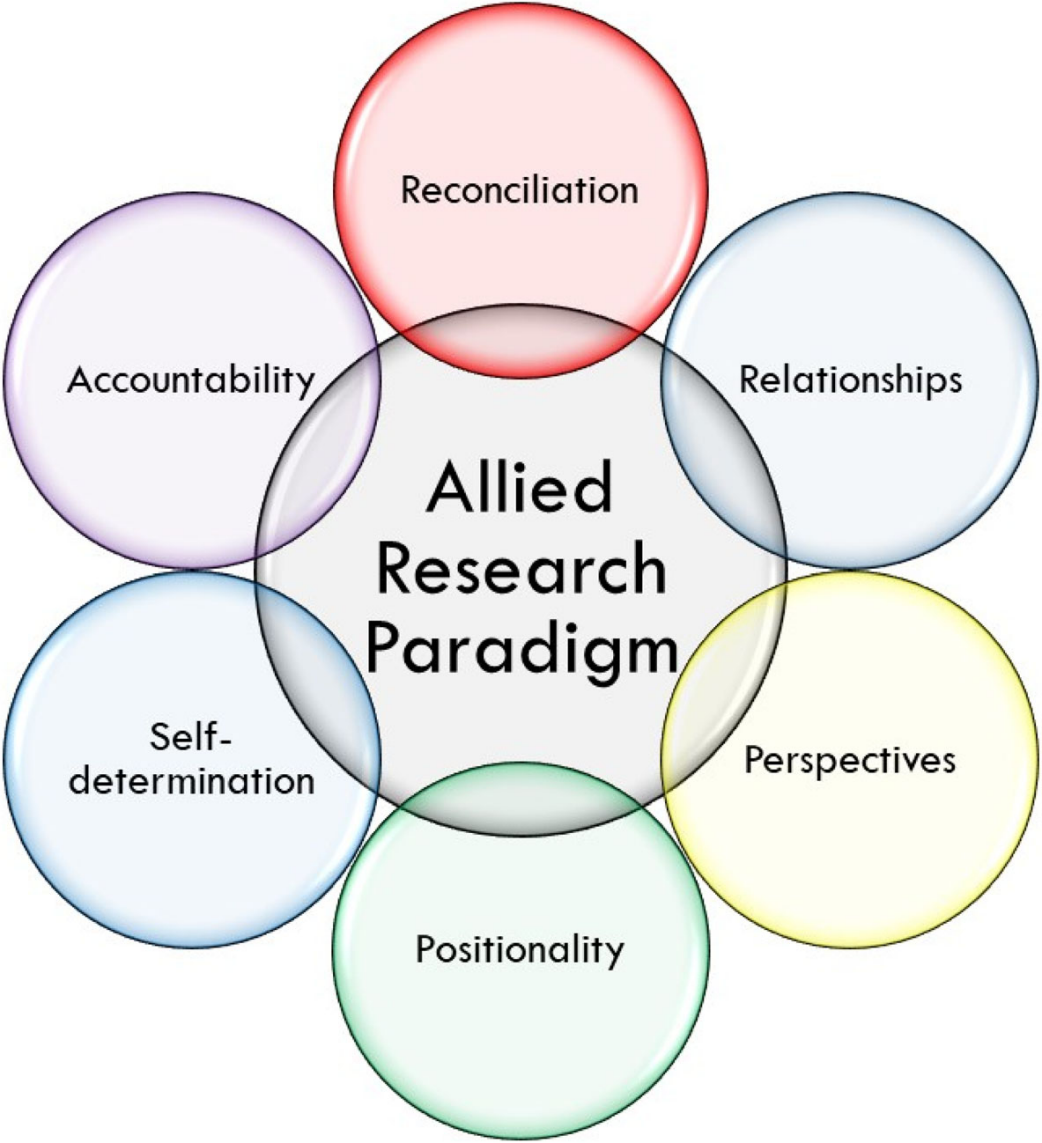
Illustration of an allied research paradigm for epidemiology research with Indigenous peoples This figure depicts six important principles of an allied research paradigm for epidemiology research with Indigenous populations. The principle of reconciliation urges researchers to build their own path to reconciliation through understanding past and present harms and committing to do research ‘in a good way.’ The principle of relationship highlights the paramount importance of forming meaningful and sustainable relationships in research. The principle of perspective asks researchers to value Indigenous knowledges and worldviews and also to ensure the research is seen as valuable from the perspective of the impacted communities. The principle of positionality implores researchers to understand their positionality and how it influences their research. The principle of self-determination reminds researchers that to act in allyship requires commitment to support the self-determination of Indigenous communities in health research. The principle of accountability emphasizes that the researcher must remain accountable to their research partners, participants and the knowledge generated long after the research activities have concluded.

I do not see myself as a researcher with expertise in Indigenous health. It is not my place to research Indigenous peoples. There are many Indigenous academic groups and researchers that can lead epidemiology studies related to Indigenous health that are driven by Indigenous research priorities. I will support Indigenous research if I am invited, but I do not want to build my career on doing research that may not be wanted or could be used to further oppress Indigenous populations. I have recently moved to a new region in Northwestern British Columbia which is on the territory of the *Tsimshian* peoples. As a recent settler in this area, I am just beginning to build relationships that I hope to evolve over my lifetime. As such, I am writing based on my prior experiences in Toronto and Vancouver, Canada I have learned many lessons that are important for allied researchers and I share them with you below. I have identified six principles that are the most important for me when I think about engaging with Indigenous communities: reconciliation, relationships, perspective, positionality, self-determination and accountability (Fig. 1). I have placed these principles within a circle so there is no hierarchy. They are each important in different ways and also are inter-related and cannot thrive without each other. I believe that including these principles in work that I do will help to make my research more culturally safe and scientifically valid [48]. I have also included a table containing examples of how these principles can be applied, using the *Building Bridges* project as a model (Table 1) [49]. *Building Bridges* was a community-based research project that I had the opportunity to be involved in during my medical residency. This project aimed to make HIV cohort data accessible to Indigenous community members and resulted in three analyses based on research priorities identified by Indigenous peoples living with HIV in Canada [49–52].

**Table 1:** Applying an allied research paradigm to epidemiology using the Building Bridges study as an example

Important principles in an allied research paradigm	Examples of how these can be applied in epidemiology research
Reconciliation	The research team met with Indigenous stakeholders prior to conducting the research to discuss how to mitigate potential harms of the research and create a safe space for participants.
Relationship	This research evolved out of existing relationships among settler researchers and Indigenous people living with HIV and these relationships were strengthened during and after the research.
Perspective	This research supported Indigenous people living with HIV to develop research questions which were then answered through cohort analyses.
Positionality	Researchers participated in research alongside Indigenous people living with HIV. They shared information about themselves and why they wanted to be a part of this research. All involved in the study became participants in a way and researchers in a way.
Self-determination	Indigenous team members were supported to write manuscripts and present research findings.
Accountability	A celebration and feast was held at the end of the study to share findings with all participants and celebrate what had been accomplished.

This table uses the Building Bridges study [49] to provide an example of how the principles of an allied research paradigm can be applied to epidemiology research

I have identified these as important through my personal journey and approach them from the perspective of a settler. They also echo many principles identified by Indigenous scholars [7, 40] and are supported explicitly or implicitly in the United Nation Declaration on the Rights of Indigenous Peoples [53]. Every journey is unique and others may find other principles they wish to include. Health researchers should also be aware of the Competencies for Indigenous Public Health, Evaluation and Research which have been articulated by a transnational coalition of Indigenous scholars [54]. In addition, my journey is ongoing. Ten years ago, I would have identified other principles and I suspect that over the next ten years my view will continue to shift, enriched by my experiences and the relationships that I form along the way. This paradigm is also unique to the context of settler colonialism, as my journey has been situated in Canada where Indigenous people have been and continue to be inordinately impacted by settler colonialism. An allied research paradigm with different groups or colonial histories may share similarities but will also include unique aspects specific to that context.

#### Begin your own path to reconciliation

The Truth and Reconciliation Commission (TRC) of Canada was established as a response to the state-sanctioned forcible removal of Indigenous children from their families, which has been described as a form of genocide leading to a legacy of health disparities [55]. The TRC has brought the term ‘reconciliation’ to the forefront of public discourse in Canada, but it is far from being embedded into our collective identity as Canadians. Many, including myself, are still struggling to understand how to embrace a path of reconciliation. The TRC describes reconciliation as being “about coming to terms with events of the past in a manner that overcomes conflict and establishes a respectful and healthy relationship among people, going forward” [55].

It is important to recognize that reconciliation can have very different meanings for different people. Conflicting views of reconciliation exist, including those of the settler state or academic institutions and those of many Indigenous communities [55]. Indigenous activists and scholars such as Taiaiake Alfred and Arthur Manual present critical views of the term ‘reconciliation,’ suggesting that it can be employed as another tool to further colonial agendas and that reconciliation from an Indigenous perspective must be tied to Indigenous land rights, sovereignty and preservation of languages and culture [56]. If we are to be allied researchers, we must develop our own understanding of reconciliation that is shaped by our experiences and the relationships we hold with Indigenous communities and transform this understanding into action.

In health research, reconciliation can include understanding the negative health impacts of residential schooling and other acts of colonialism along with a commitment to dismantle the colonial structures that continue to fuel health inequities. A path to reconciliation for health researchers requires an understanding of how health research itself has and continues to threaten Indigenous populations, for example through deficit-based approaches to Indigenous health. Tuhiwai Smith [39], criticizes existing research on Indigenous peoples for how it has been used as a tool by western researchers to exert dominance over Indigenous populations. She describes research as “a powerful intervention, even if carried out at a distance, which has traditionally benefited the researcher, and the knowledge base of the dominant group [39].” Racist assumptions of Indigenous inferiority can influence epidemiology research by guiding research question design, covariate selection and data interpretation. Findings from studies that portray Indigenous peoples as deficit have been used to dispossess Indigenous peoples of their land, children and culture [55]. If we are to be allied researchers, we must educate ourselves about this reality and recognize that well-intentioned studies do not necessarily lead to improved health and wellness of the populations we wish to engage with in research. We must carefully consider how the intersection of social and political factors contribute to environments of disease risk and ensure this is reflected in research design [33, 57, 58]. Research, if done properly and collaboratively, can then become an act of reconciliation as strong and meaningful relationships are built among allied researchers and Indigenous communities.

Drawing on Shawn Wilson’s relational accountability, reconciliation also requires the nurturing of robust and longstanding relationships among Indigenous communities, health professionals and researchers. Reconciliation respects and fosters Indigenous self-determination in health and health research [55] and allied researchers can turn to the Ownership, Control, Access and Possession (OCAP™) principles of the First Nations Information Governance Centre for guidance [59]. Inuit and Métis organizations have also developed their own guides to research engagement [60, 61]. In addition, specific Indigenous communities may have developed their own protocols for research and self-determination in research and it is the responsibility of non-Indigenous researchers to be respectful and adherent of local protocols for conducting research.

#### Build and maintain relationships

Consistent with community-based participatory methodologies, research involving Indigenous peoples must be built on meaningful, reciprocal relationships with the communities whose data are being collected and a substantial amount of time must be dedicated to building these relationships [9, 26]. Shawn Wilson’s [7] description of relational accountability can guide allied health research. Allied researchers must develop relationships with research participants, community members and their research and must remain accountable to these relationships for as long as they exist. We have a responsibility to do research in the best interest of the research participants, and to follow through on the findings so they can be of direct benefit to the community. We must also allow for fluidity in relationships. In my own experience, I have seen a researcher-participant relationship evolve to one where I consider the ‘participant’ to now be a colleague, mentor and friend. Research relationships, as they evolve, need to be based on reciprocity [43]. Johnson and Madge articulate that reciprocity, “while predicated on both acts of giving and receiving, is motivated by giving: not giving as charity, but giving as honouring [20].” While the relationships that the researcher forms with others and with the research itself are crucial, an often overlooked relationship is the relationship that the researcher forms with themselves. Ongoing reflexivity as well as self-care are needed to ensure that the researcher has a healthy relationship with themselves and this relationship cannot be forgotten.

#### Honour the value of indigenous perspective

If we are to be allied researchers, we need to first confront our own lack of experience. As Mi’kmaq scholar, Cyndy Baskin, poignantly argues, one can never be an expert in a community that they do not belong to. By valuing Indigenous perspectives, research can be both more ethical and more valid [43]. Leung argues that epidemiologists who engage community members in research design and analysis have found that their community partners bring a “sophisticated understanding of the connections between problems and issues identified which might otherwise have been completely missed [32].”

This also means that allied researchers must recognize that our worldviews are not the only ones that exist and that meaningful research relationships can only occur if we are able to value non-dominant perspectives and understandings of knowledge [7]. For example, Mohawk scholar, Marlene Brant Castellano, describes how many Indigenous peoples seek knowledge from traditional teachings (knowledge that has been passed down through generations), empirical observation (knowledge from observation over time from multiple perspectives) and revelation (knowledge that is spiritual in origin) [62]. For cultural safety to exist in allied research, Indigenous knowledge, values and epistemologies must be seen as equal and complementary to dominant ways of knowing [48].

Indigenous peoples must provide leadership in all aspects of the research process, including hypotheses development, research question generation, methods selection and interpretation [25, 26, 41, 48]. A key principle of decolonizing methodologies such as a *Kaupapa Māori* framework as described by Tuhiwai Smith includes power sharing within research by allowing ‘the researched’ to identify the research priorities, important outcomes, relevant confounders and appropriate ways to measure variables [39]. This principle is an important part of an allied research paradigm.

Tuhiwai Smith explains the importance of full engagement of Indigenous peoples in research:

"When indigenous peoples become the researchers and not merely the researched, the activity of research is transformed. Questions are framed differently, priorities are ranked differently, problems are defined differently, people participate on different terms [39]."

This highlights the value of Indigenous leadership in research and also the importance of research that is addressing priorities of importance to Indigenous communities.

I also look critically on my own prior publications on Indigenous outcomes in HIV cohort studies, all of which were conducted collaboratively with Indigenous partners who are leaders in HIV. In a cohort analysis on late diagnosis of HIV among Indigenous peoples, I worked with Indigenous co-investigators who provided guidance into the study, but their role was supportive and they were asked to participate after the research question had been established. A subsequent analysis on coping strategies and support was conducted based on a research question of one of our Indigenous co-investigators. A third analysis, of a similar cohort, engaged a group of Indigenous women living with HIV throughout the entire research process and the manuscript was drafted as two of these women and I shared a meal together in my home. I can see how my understanding of meaningful involvement has evolved and I look back on some of my earlier work with regret because what I considered meaningful at that time feels tokenistic to me based on my current appreciation of meaningful engagement. All along this journey, I am so grateful for my Indigenous colleagues who have taken the time to mentor me and have treated me with understanding and compassion.

#### Identify your positionality

Throughout the literature, Indigenous and critical feminist scholars emphasize the importance of stating your positionality. The importance of positionality, a manifestation of social position can be understood as described by Walters and Anderson who explain that “we do, live, and embody social position, and as researchers, it covertly, overtly, actively, and continuously shapes how we do, live and embody research practice [47].” Researchers should explicitly describe who they are and how their life circumstances shape their research [34]. We should offer a critical discussion on the implications of our own subjectivities and motives and the relationship that these have with the research process and outcomes. How we conceptualize and conduct research is influenced by our personal and professional positionalities and acknowledging and discussing our positionality strengthens rather than discredits the research [32].

In addition, as researchers, we often hold other roles, such as healthcare provider or colleague and it is important to understand our own multiplicity of roles, how they interact and even how they conflict when we do our research [41]. Our roles and social positions are also inseparably bound to power relations. For me personally, I must always be aware of how my role as a specialist physician in an underserved region will change how people engage in research with me. I must ask myself why people choose to collaborate with me or participate in research that I am involved in. Do they have interest in contributing to the knowledge and action that are expected products of the research? Does my social position give them an unrealistic expectation of the impact the research will have? Do they perceive that research engagement will influence how I provide clinical care to them and their families?

#### Support indigenous self-determination

The role of the allied researcher is evolving within the field of Indigenous health research. In the past, this has been dominated by generally well-intentioned non-Indigenous researchers, sometimes working in collaboration with Indigenous communities. As more Indigenous epidemiologists and quantitative researchers take leadership roles in this field, allied researchers can and should create space for Indigenous self-determination in epidemiology. I was once asked to act as a student data analyst for the Indigenous working group of a cohort study. As the study progressed, the group indicated that they were hoping for an Indigenous student to hold that role and they now had someone in mind. My initial reaction was to be offended and feel as though my contributions were not valued. As I reflected, I realized that the best thing that I could do for the project was to stop occupying the space that should be filled by an Indigenous student and to support her in her new role. I could continue to contribute where asked, but needed to be mindful not to impose my ‘help’ on the project.

Decolonization of academic institutions includes more than just increasing numbers of Indigenous faculty members and students. This must occur in parallel with an increased acceptance of Indigenous methodologies. There can be a role for Indigenous methods and methodologies in epidemiology, for example, by using sharing circles or land-based methods to identify quantitative research questions and interpret the findings. Indigenous knowledge sharing tools such as oral histories and story-telling can be used to inform research design, variable selection and model building. I have been fortunate to have been a team member in the *Building Bridges* study [49] which is an example of how Indigenous methodologies can work synergistically with epidemiology methods. Even if we do not use Indigenous methodologies, we must support our Indigenous colleagues so that their scholarly work is recognized. We also must be acutely aware of whose voice we chose to privilege and cite in our own writing [63]. We can also consider models that draw from the strengths of both western scientific knowledge and Indigenous knowledge such as Two-Eyed Seeing which was first described by Mi’kmaq Elder, Albert Marshall [64].

#### Remain accountable to all your relationships

Research does not end with a peer-reviewed publication and accountability in research is essential [7, 35]. We are accountable to our research participants, our community partners, our research and ourselves. As Krieger and Zierler explain, “ultimately, we are accountable for the knowledge we produce and its effects on the public’s welfare [35].” There are many ways that we can be accountable throughout the research process. Maintaining relationships is imperative as is ensuring sustainability of these relationships. Research agreements are another way of documenting the accountability within research relationships [65]. The impact of the research must also be sustained. This can begin with sharing findings with participants [31] and using knowledge translation strategies and products that are culturally- and context-appropriate [66]. It also means that as we begin planning our research, an important consideration is how the findings will directly be used to improve the health and wellness of the studied peoples.

## Discussion and conclusions

Epidemiology is often seen as a “dirty” word among Indigenous communities because of the harm that it has and continues to cause [39]. However, I believe that it is not epidemiology itself that is the problem, but rather the way that it has been used by agents of settler states to inadvertently and intentionally oppress Indigenous peoples. The tools of epidemiology can be used to support the health and wellness of Indigenous peoples, but researchers who hope to travel down this path must do so thoughtfully and in partnership with Indigenous communities. By expanding our research paradigm beyond a purely positivist approach and broadening our perspective to be inclusive and respectful of other ways of knowing, we can begin to reconcile our quantitative methodologies with the priorities of the communities we aim to serve. By considering our own path to reconciliation, building meaningful partnerships, honouring Indigenous knowledge and perspectives, reflecting on our positionality, supporting Indigenous self-determination in research and maintaining accountability throughout and after the research process, we may become worthy of calling ourselves allies. These are some of my reflections based on my current and past experiences, but as I grow as a researcher and as a person, I expect them to mature. As Shawn Wilson attests, “if research doesn’t change you as a person, then you aren’t doing it right [7].”

## Abbreviations

AIDS: Acquired immune deficiency syndrome
CBPR: Community-based participatory research
HIV: Human immunodeficiency virus
OCAP: Ownership, control, access, possession
TRC: Truth and Reconciliation Commission

## Author’s information

DJ is a settler physician in the speciality of General Internal Medicine. She practices in the community of Terrace in northern British Columbia, Canada. She is also a PhD candidate in Clinical Epidemiology and Health Care Research in the Institute for Health Policy, Management & Evaluation at the University of Toronto.
